# Vascular Regulation of Developmental Neurogenesis

**DOI:** 10.3389/fcell.2022.890852

**Published:** 2022-04-29

**Authors:** Johanna Vogenstahl, Marta Parrilla, Amparo Acker-Palmer, Marta Segarra

**Affiliations:** ^1^ Neuro and Vascular Guidance Group, Buchmann Institute for Molecular Life Sciences (BMLS) and Institute of Cell Biology and Neuroscience, Frankfurt am Main, Germany; ^2^ Max Planck Institute for Brain Research, Frankfurt am Main, Germany; ^3^ Cardio-Pulmonary Institute (CPI), Frankfurt am Main, Germany

**Keywords:** neurogenesis, blood vessels, neurovascular communication, hindbrain, forebrain

## Abstract

Evolutionary studies indicate that the nervous system evolved prior to the vascular system, but the increasing complexity of organisms prompted the vascular system to emerge in order to meet the growing demand for oxygen and nutrient supply. In recent years, it has become apparent that the symbiotic communication between the nervous and the vascular systems goes beyond the exclusive covering of the demands on nutrients and oxygen carried by blood vessels. Indeed, this active interplay between both systems is crucial during the development of the central nervous system (CNS). Several neural-derived signals that initiate and regulate the vascularization of the CNS have been described, however less is known about the vascular signals that orchestrate the development of the CNS cytoarchitecture. Here, we focus on reviewing the effects of blood vessels in the process of neurogenesis during CNS development in vertebrates. In mammals, we describe the spatiotemporal features of vascular-driven neurogenesis in two brain regions that exhibit different neurogenic complexity in their germinal zone, the hindbrain and the forebrain.

## Introduction

In the course of phylogenic evolution, the nervous system precedes the appearance of the vascular system. The first organisms that developed a primitive nervous system were diploblasts, i.e., cnidarians (jellyfish, anemones, corals) and ctenophores (jelly comb) (Hartenstein and Stollewerk, 2015; Arendt et al., 2016). These animals exhibit a diffuse nerve net and lack a vascular system, as oxygen and nutrient demands can be met by simple diffusion (Monahan-Earley et al., 2013). Millions of years later, more complex organisms emerged, the triploblasts, which have bilateral symmetry and a tubular nervous system. With their increased complexity and body size, it became necessary to develop a circulatory system to transport fluid throughout the whole organism. This incipient circulatory system evolved into a blood vascular system.

Interestingly, the nervous system also precedes the vascular system during embryogenesis. The neural tube, the origin of the central nervous system (CNS) in bilaterian animals, is formed by neuroepithelial cells derived from the ectoderm (Hartenstein and Stollewerk, 2015) and is avascular *ab initio* (James et al., 2009). In vertebrates, neuroepithelial cells that form the neural tube initially undergo symmetric divisions in synchrony with interkinetic nuclear migration [see reviews (Miyata, 2008; Taverna and Huttner, 2010)]. This process of cell proliferation serves to amplify the pool of progenitor cells before the onset of neurogenesis (Subramanian et al., 2017). Next, from embryonic day (E) 10.5 onwards in mouse (Haubensak et al., 2004), neuroepithelial cells divide asymmetrically to generate radial glial cells (RGCs). RGCs are neuronal progenitor cells with cell-renewal and neurogenic capacity (Malatesta et al., 2000; Noctor et al., 2001), representing the cellular source for almost all neural lineages of the CNS [see review (Gotz and Huttner, 2005)]. RGCs are morphologically similar to neuroepithelial cells, although they are more elongated [see review (Arai and Taverna, 2017)]. Both cell types exhibit apical-basal polarity and span their processes across the neural tube. The apical end-feet contact the lumen of the neural tube whereas their basal fiber anchors at the pial surface. Moreover, RGCs are not only the source of neuronal progenitors and neurons, but the basal processes of these cells are also used as scaffolds for the migrating newborn neurons (Rakic, 1971; Nadarajah and Parnavelas, 2002).

Neurovascular interactions happening before the onset of neurogenesis have not been described; however, following the closure of the neural tube, angioblasts (endothelial progenitor cells) are recruited from the pre-somitic mesoderm to surround the neural tube with a vascular mesh, termed perineural vascular plexus (PNVP) (Kurz et al., 1996; Ambler et al., 2001). These primitive vessels then sprout radially into the neural tube developing a ramified vascular network, called intraneural vascular plexus (INVP). In mouse, PNVP establishment occurs between E8.5 and E10, followed by the emergence of INVP at around E10.5 [see reviews (James and Mukouyama, 2011; Segarra et al., 2019)], therefore the onset of neurogenesis is timely harmonized with neural tube vascularization. Moreover, initial experiments in chick embryos showed a stereotypical pattern in the formation of the INVP (Feeney and Watterson, 1946), indicating that sprouting angiogenesis into the primitive neural tube is guided by neural-derived cues. This hypothesis was demonstrated later, and neural-derived vascular endothelial growth factor A (VEGF-A) was shown to be one of the major signals orchestrating neural tube vascularization (Hogan et al., 2004; James et al., 2009).

In the developing CNS, vessels establish an intimate relationship with RGCs. On one side, the vascular sprouts of the INVP align with the RGCs processes (Noctor et al., 2001; Gerhardt et al., 2004; Ma et al., 2013). On the other side, the long basal processes of the RGCs contact the pial surface irrigated by vessels of the PVNP while RGC somatas and their short apical processes lie on the ventricular side of the neural tube, where the INVP further develops and forms the periventricular plexus (PVP) [see review (Peguera et al., 2021)]. Developmental neurogenesis is a multi-step process that encompasses several waves of cell division, followed by migration and differentiation of neuroblast cells, and culminates with the integration of mature neurons into the neural circuit (Bjornsson et al., 2015). The vasculature, which intermingles and develops symbiotically with the growing CNS, may help to guide and coordinate the different stages of neurogenesis.

Communication between vessels and neural cells is bidirectional. Several studies have deciphered which neuronal cues regulate CNS vascularization [reviewed in (Paredes et al., 2018)], but less is known about the instructive role of endothelial cells in modulating neuronal processes. Emerging data reveal that the vasculature plays a functional role in CNS development, including embryonic neurogenic niches. In this review, we focus specifically on the influence of the vasculature along the neurogenic journey and its potential control of neuroblast cell division, migration and differentiation during CNS development.

## Lessons From *in Vitro* Systems

Co-culture systems of neural stem cells (NSCs) and endothelial cells have helped to demonstrate the influence of the endothelium on the neurogenic niche (Shen et al., 2004; Sun et al., 2010; Vissapragada et al., 2014). Most of these studies suggest that endothelial cells induce the proliferation of NSCs to increase the undifferentiated pool of neurons. For example, Shen et al. showed that soluble factors secreted by endothelial cells promote the symmetric division of NSC, whereas NSC undergo differentiation in the absence of endothelial cells (Shen et al., 2004). Also, an enhanced proliferation of NSC was observed when neuronal progenitors were co-cultured with embryonic brain endothelial cells from PVP origin, therefore reproducing the embryonic neurogenic niche *in vitro* (Vissapragada et al., 2014). Interestingly, variations in the co-culture conditions could trigger different effects on the neuronal progenitors: whereas soluble factors led to NSCs self-renewal, direct contact with the endothelium promoted neuronal progenitor cell differentiation (Gama Sosa et al., 2007). This divergent response provides an indication of the complexity of the neurogenic dynamics *in vivo* and the diversity of signaling mechanisms that may be derived from the interplay with the endothelium.

## Vascular-Guided Neurogenesis in Non-Mammalian Vertebrates

Non-mammalian models have been crucial in the study of neuronal development (Marder, 2002). In zebrafish, the avascular mutant *cloche* is a powerful model to investigate the neurovascular interactions during development. *Cloche* zebrafish have a dysfunctional heart, which impairs blood circulation, as well as lack blood cells and most of the vasculature (from 20 to 26-somite stage) (Stainier et al., 1995; Liao et al., 1997). In the hindbrain, blood vessels develop in close association with subsets of neuronal clusters in early stages (48–72 h post-fertilization, hpf), but the absence of vessels in *cloche* has no impact on local neurogenesis (Ulrich et al., 2011). However, in other brain regions at the same developmental stage, such as the cerebellum’s upper rhombic lip and the optic tectum, the axonal scaffolds were reduced in *cloche*, presumably because their development requires blood flow and/or signals from the surrounding vessels (Ulrich et al., 2011). Interestingly, Taberner et al. demonstrated using *cloche* mutants that blood flow is necessary for cranial sensory neural differentiation (54–72 hpf) in the statoacoustic ganglion via activation of genes related to oxygen metabolism (Taberner et al., 2020). Besides blood flow, blood-borne signals may potentially influence the neurovascular niche during development. Indeed, in *Xenopus laevis*, neuronal progenitors that line the ventricle and extend their radial processes to establish contact with the pial surface are able to internalize circulating dextran through their end-feet (Lau et al., 2017). However, no relationship was found between neural progenitor’s end-feet-blood vessel contacts and their cell division rate.

Direct contact between endothelial cell and neural progenitor also seems to regulate neuronal development. In early cranial sensory neurogenesis of the statoacustic ganglion in zebrafish (30–36 hpf), direct interaction of endothelial cells and neuronal progenitors regulate their proliferation (Taberner et al., 2020). Thus, loss of vasculature in *cloche* correlates with a neuroblast increase in this region, indicating that endothelium-neuroblast contacts negatively regulate neurogenesis by keeping neuroblasts quiescent. Those contacts are mediated by the endothelial cell cytoneme, a thin actin-based cellular extension specialized for cell-cell communication that binds to cranial sensory neuroblasts and communicates via Dll4-Notch signaling pathway (Taberner et al., 2020).

In zebrafish retina, *cloche* mutants also lack vasculature and show prominent defects in cell proliferation, survival, organization and differentiation (30–72 hpf) (Dhakal et al., 2015). These defects in retinogenesis were independent from hypoxia, but *cloche* mutants did not allow to differentiate the role on retinal neurogenesis between endothelial cells, blood-borne factors and/or circulating blood cells. To address this, Dhakal et al. used three different mutant models characterized by: 1) absence of endothelial cells, 2) lack of blood flow and 3) no erythroid lineage cells. This strategy revealed that factors derived directly from endothelial cells are major key players in cell proliferation and differentiation in the retina; although circulating factors might also play a role in these processes (Dhakal et al., 2021). Interestingly, the ciliary marginal zone, where the retinal neurogenic niche resides, is severely affected in the absence of endothelial cells (Dhakal et al., 2021). Consistent with this, blood vessels associated with retinal stem cells in the ciliary marginal zone were shown to be required to maintain them in proliferative stages (Tang et al., 2017). In the developing rat retina, *in vitro* and *in vivo* studies also support that endothelial cells regulate the cell self-renewal of retinal progenitor cells via the epigenetic regulator *Hmga2* (Parameswaran et al., 2014).

Taken together, all data suggest that the role of the vasculature in neurogenesis is very variable depending on the region and developmental stage. Blood vessels may govern diverse mechanisms leading to different responses, from the balance between proliferation and quiescence to differentiation.

## Contribution of the Vasculature to Developmental Neurogenesis in Mammals

Vascular regulation of developmental neurogenesis has been studied in the neurogenic niches of the hindbrain and the forebrain in the embryonic mouse (Karakatsani et al., 2019). The hindbrain gives rise to the cerebellum, pons, and medulla oblongata; whereas the forebrain differentiates into the diencephalon and the telencephalon, which generates neurons that populate the vast neocortex and the subcortical structures (such as hippocampus and basal ganglia).

The hindbrain is the most functionally and developmentally conserved region in the evolution of the vertebrate brain (Krumlauf and Wilkinson, 2021). In contrast, the evolution of the neocortex across vertebrates is variable and shows differences in tissue structures, for example number of neocortical layers (Briscoe and Ragsdale, 2019). Cortical neurogenesis is evolutionary conserved in mammals; however, the cerebral cortex is also characterized by a wide variability in volume and folding complexity across species. This could be related to a prolonged neurogenic period that correlates with the duration of gestation, exposing the developing neocortex to maternal environment for a longer period of time. This includes a whole variety of circulating factors, such as hormones, that are delivered by the blood vessels and the cerebrospinal fluid system and potentially influence neurogenesis (Montiel et al., 2013; Stepien et al., 2021).

### Neurogenesis in the Developing Hindbrain

**FIGURE 1:**
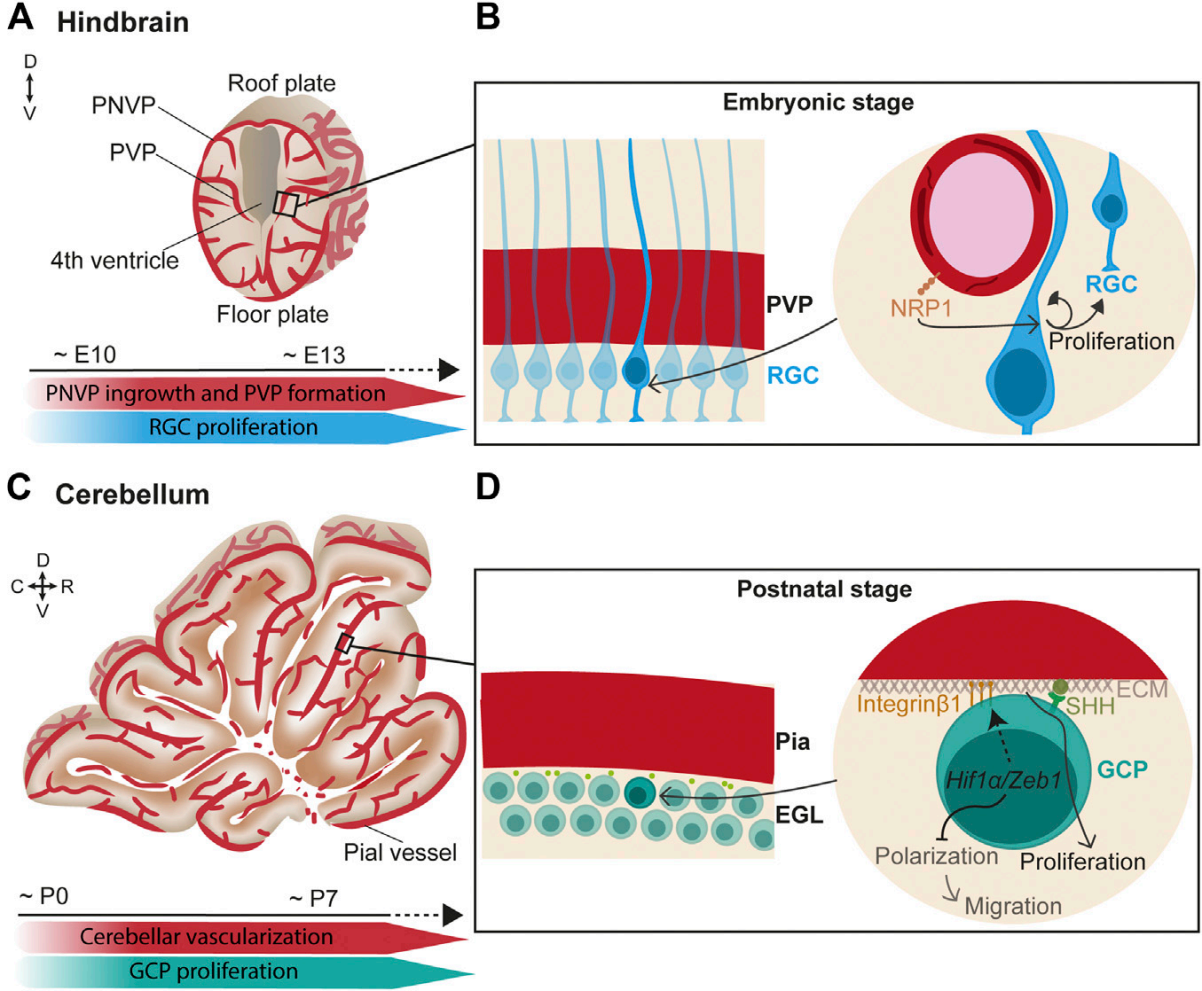
Endothelial cell signaling regulates neurogenesis in the hindbrain/cerebellum. **(A)** (Top) Scheme of a coronal view of the hindbrain at the embryonic stage. Vessels from the perineural vascular plexus (PNVP) ingress into the neural tissue in a perpendicular manner towards the ventricle where they form the periventricular vascular plexus (PVP). Then, lateral sprouts emerge and form a more complex vascular network. (Bottom) Timeline of concurrent hindbrain embryonic vascularization and radial glia cells (RGC) proliferation. **(B)** (Left) Closer view of the ventricular zone containing a layer of radial glial cells (RGC) that extend their basal fibers towards the pial surface and the apical short processes contact the ventricle. (Right) RGC basal fibers directly contact the PVP vessels. In this scenario, endothelial NRP1 signaling maintains RGC proliferation through a mechanism independent of hypoxia and VEGF. **(C)** (Top) Scheme of a sagittal view of the cerebellum at the first postnatal week. (Bottom) Timeline of concurrent cerebellar postnatal vascularization and granule cell progenitors (GCP) proliferation. **(D)** (Left) Granule cell progenitors (GCP) reside under the pial vessels in the cerebellum and form the external granule layer (EGL). (Right) In hypoxic conditions, *Hif1α/Zeb1* favors the GCP attachment to the extracellular matrix (ECM) of the pial surface through Integrin β1 expressed at the GCP membrane. In parallel, *Hif1α/Zeb1* inhibit GCP polarization and subsequent migration. Components of the pial ECM (laminins) enhance SHH signaling which, in turn, promotes GCP proliferation.

In the hindbrain, vessels from the PNVP (which later becomes the meningeal vasculature) penetrate radially into the neural tissue towards the ventricular zone, where they turn and anastomose to form the PVP at around E10 and onwards (Figure 1A) (Fantin et al., 2013). Subsequently, lateral sprouts emerge and anastomose to form a more complex plexus.

Interestingly, direct neurovascular contacts are described in the germinal zone of the hindbrain surrounding the ventricle. Confocal microscopy and 3D reconstructions suggested that hindbrain neural progenitor processes and end-feet directly contact PVP blood vessels (Tata et al., 2016). In support of a neurovascular communication, a spatiotemporal congruency was found between the sprouting of vessels in the PVP and the peak of neural progenitor proliferation. Moreover, endothelial deletion of Neuropilin1 (NRP1), a co-receptor of VEGF-A, resulted in premature differentiation of neural progenitor independent of VEGF signaling and hypoxia (Figure 1B) (Tata et al., 2016). This suggests that PVP vasculature directly regulates neurogenesis.

The hindbrain is the premise of the cerebellum, which mostly develops postnatally. In the cerebellum, glutamatergic neurons called granule cells originate in the upper rhombic lip. During embryonic development, granule cells proliferate and migrate anteriorly to cover the entire dorsal cerebellar surface, where they create a postnatal secondary neurogenic niche, the external granule layer (EGL) (Consalez, 2021). This migration process is mediated by the interaction of C-X-C motif chemokine 12 (CXCL12), expressed by the leptomeninges, and its receptor CXCR4, expressed by the migrating progenitor cells (Zhu et al., 2004; Hagihara et al., 2009). Later, CXCL12 signaling is suggested to arrest neuronal progenitors at the pial surface in the EGL (Zhu et al., 2004; Vilz et al., 2005; Consalez, 2021). At perinatal stages, the EGL actively proliferates in mice until the third postnatal week. During the first postnatal week (Figures 1C,D), the cerebellar cortex is poorly vascularized, resulting in low O_2_ tension that increases expression of the hypoxia-inducible factor *Hif1α* (Kullmann et al., 2020). HIF1α on the one hand, negatively regulates the partitioning-defective (*Pard*) gene complex via *Zeb1*, which prevents granule cell polarization and consequent migration and, on the other hand, promotes the attachment of the proliferating granule cell progenitors to the pial extracellular matrix via Integrin β1, which keeps them in the germinal zone (Figure 1D) (Kullmann et al., 2020). Moreover, components of the extracellular matrix on the pial side of the EGL, particularly laminins, enhance the response to sonic hedgehog (SHH), the best-studied morphogen that induces granule cell proliferation (Pons et al., 2001; Consalez, 2021) (Figure 1D). As cerebellar vascularization progresses, *Hif1α* expression is downregulated, and granule cells can detach from the pia and prepare for migration (Kullmann et al., 2020). Granule cells extend their axons while migrating. In this process, the interaction of Discoidin domain receptor 1 (DDR1) expressed in the granule cells with collagen secreted at the pial surface is essential for their axonal formation (Bhatt et al., 2000).

All in all, although relief from hypoxia is an important factor controlling progenitor cell division, other vascular-mediated signaling pathways directly contribute to hindbrain/cerebellum neurogenesis.

### Neurogenesis in the Developing Forebrain

Located on the edge of the telencephalic lateral ventricles, RGCs divide symmetrically or asymmetrically to expand the pool of progenitor cells, giving rise to either two RGCs or one RGC and one intermediate progenitor cell (IPC), respectively (see review (Taverna et al., 2014)). Progenitor cells continue to divide asymmetrically to give rise to neurons. The forebrain germinal zone is layered in two: the ventricular zone (VZ) where RGC somatas reside and the sub-ventricular zone (SVZ), above the VZ, where the newly born IPC accumulate from E12.5 (Paridaen and Huttner, 2014; Bjornsson et al., 2015). In some species (e.g., humans), the SVZ highly amplifies the pool of progenitors and is considered to be the evolutionary basis for neocortex expansion. Dorsal and ventral telencephalon give rise to excitatory and inhibitory neurons respectively, and both of these telencephalic regions exhibit a VZ and SVZ.

**FIGURE 2:**
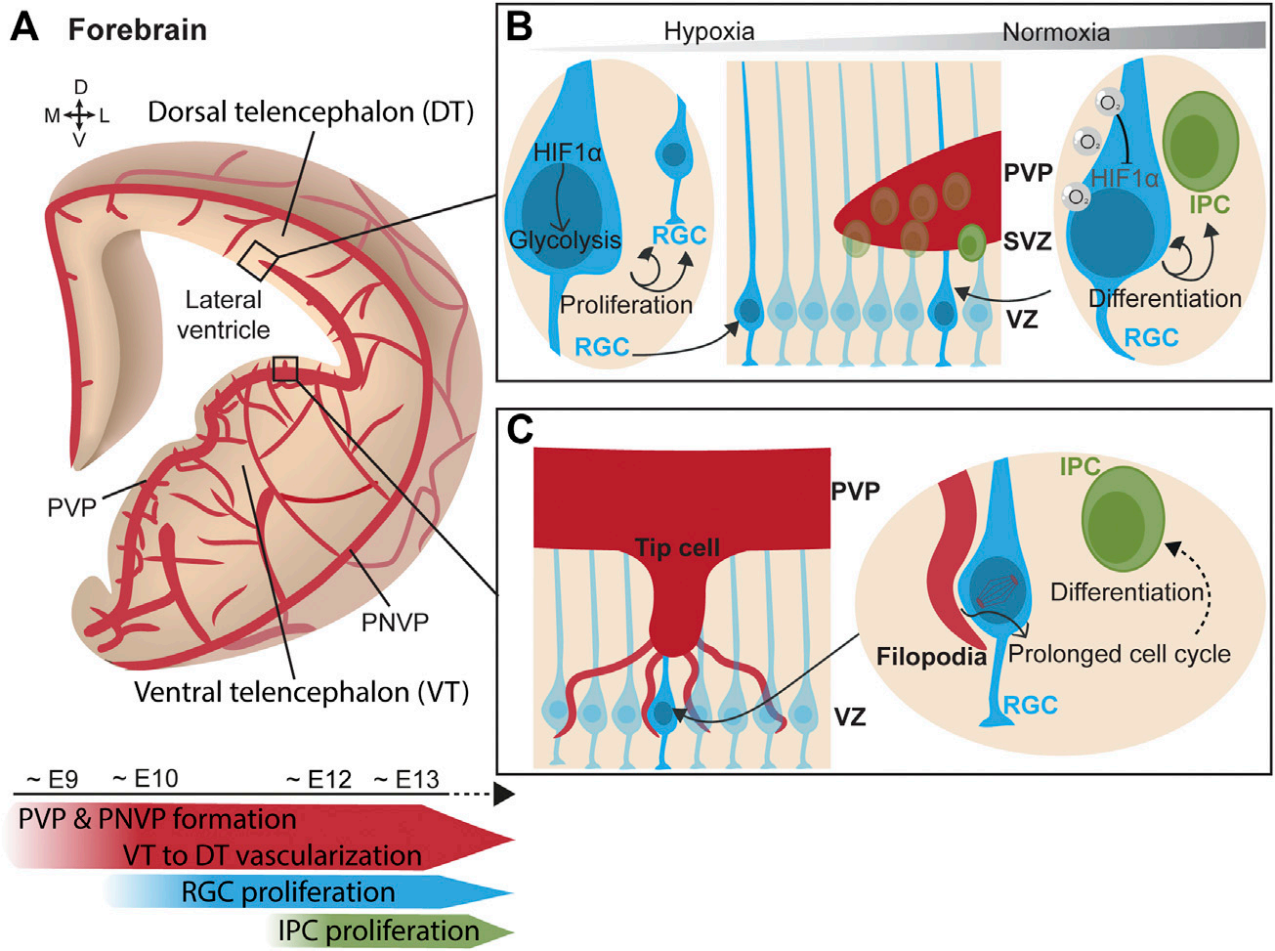
Vasculature influences neurogenesis in the forebrain. **(A)** (Top) Scheme of a coronal view of the forebrain at the embryonic stage, showing its vascularization from the perineural vascular plexus (PNVP) and the periventricular vascular plexus (PVP) in a progressive manner from the ventral telencephalon towards the dorsal telencephalon. (Bottom) Timeline of concurrent telencephalic embryonic vascularization and neurogenesis. **(B)** (Middle) Ventricular zone (VZ) contains a layer of radial glial cells (RGC), which generate intermediate progenitor cells (IPC) that form the subventricular zone (SVZ). (Left) In a poorly-vascularized and hypoxic environment, RGCs express HIF1α which triggers glycolysis and RGC proliferation. (Right) As oxygenation progresses with increased vascularization, RGC adapt to hypoxia relief. Oxygen supply from blood vessels mediates HIF1α degradation and promotes RGC asymmetric division generating IPCs. **(C)** (Left) Tip cells from ingrowing PVP blood vessel extend filopodia that directly contact RGC somatas in the VZ. (Right) Endothelial cell filopodia adhere to RGC. This direct contact prolongs the mitotic phase of cell division in RGC and favors early neuronal differentiation.

Simultaneously to the neurogenic process, forebrain vascularization starts ventrally and progressively extends towards the dorsal forebrain (Lange et al., 2016; Karakatsani et al., 2019; Puelles et al., 2019) (Figure 2A). Vessels grow from the PNVP towards the ventricle following a spatiotemporal pattern. Penetrating vessels invade the ventral forebrain already at around E10.5 whereas sprouts in the dorsal region are delayed about 1 day (Lange et al., 2016; Segarra et al., 2018). Unlike the hindbrain, an additional angiogenic source vascularizing the PVP has been identified in the forebrain. The PVP vascularization originates from a basal vessel at the telencephalic floor that branches from the basal ganglia primordium (Vasudevan et al., 2008). This vascular plexus encompassing the ventricle begins in the ventral telencephalon around E9 and progresses ventral-to-dorsal and lateral-to-medial between E10-E11 towards the dorsal telencephalon, merging simultaneously with the penetrating sprouts from the PNVP (Vasudevan et al., 2008). Confocal microscopy and 3D reconstruction of telencephalon slices show that blood vessels are omnipresent in the telencephalic neurogenic niches and form a rich PVP. This has also been observed in humans, where the ventral telencephalon is vascularized at early mid-gestation (Di Marco et al., 2020). Altogether, these findings strongly suggested that blood vessels play a critical role in embryonic neurogenic niches.

Initially blood vessels were solely described as providing a nutrients and oxygen to tissues. Following this idea, the putative contribution of blood vessels to neurogenesis was first explored through the prism of oxygenation and circulating factors. Haigh et al. elegantly laid the groundwork by inducing telencephalic devascularization and hypoxia after deleting neuronal VEGF-A, resulting in a decreased neuronal proliferation in the VZ-SVZ (Haigh et al., 2003). Vascular-specific *Gpr124* KO mice exhibit hypoxia in the VZ region. The consequent induction of *Hif1α* in this mouse model was found to maintain the proliferative state of RGCs by triggering glycolysis and to inhibit their neuronal differentiation (Lange et al., 2016). In other words, in the early stages of development, the supply of oxygen through blood vessels is poor, which makes neurogenic niches highly hypoxic. Hypoxia maintains RGCs in a proliferative state and stimulates angiogenesis. When the tissue becomes more vascularized, hypoxia is alleviated and HIF1α signaling downregulated. This results in reduction of neural progenitor cell expansion and in their differentiation into IPCs (Bjornsson et al., 2015; Lange et al., 2016) (Figure 2B). This effect of hypoxia is reminiscent to the observations in the postnatal cerebellum, since in both regions the increase in vascularization regulates neuronal differentiation. While hypoxia was the first indication of a vascular contribution to embryonic neurogenesis, it became clear that the vascular system regulates neural progenitors using other strategies.

Endothelial cells are capable of secreting factors, called angiocrines, that are crucial for regulating neurogenesis. For instance, conditionally deleting *Vegf* in endothelial cells led to several developmental defects in the embryonic telencephalon (Li et al., 2013), such as: impaired angiogenesis, abnormal localization of proliferating neuronal progenitors outside of the neurogenic niche of the dorsal telencephalon, disrupted radial glia scaffold, and defective radial migration of post-mitotic pyramidal neurons. Moreover, the tangential migratory stream of inhibitory neurons towards the dorsal telencephalon was also altered by the endothelial deletion of *Vegf*, leading to focal misplacement of neurons in the marginal zone even above the pial surface, disturbed axonal tracts, and defective cortical lamination (Li et al., 2013). Reelin is a neuronal guidance cue secreted by Cajal-Retzius cells in the marginal zone of the neocortex (Tissir and Goffinet, 2003). Deletion of the Reelin effector molecule *Dab1* in endothelial cells also resulted in several defects in forebrain cytoarchitecture, such as invasion of migrating pyramidal neurons in the marginal zone, altered positioning of neurons in the cortical layers, and disrupted adhesion of RGC processes to the pial surface (Segarra et al., 2018). However, no defects were detected in the pool of neuronal progenitors in the germinal zone, suggesting that lack of Reelin signaling in the endothelial cells preferentially impacts on neurovascular interactions at the pial surface rather than at PVP vasculature. Indeed, endothelial cells from the PVP exhibit differential gene expression compared with cells from the pial surface, suggesting that the endothelium selectively guides the tangential migration of inhibitory interneurons along the pial or the periventricular streams (Won et al., 2013). Furthermore, secretion of the neurotransmitter Gamma-Aminobutyric Acid (GABA) by endothelial cells contributes to long-distance tangential migration of inhibitory interneurons from the ventral telencephalon to their final position in the neocortex (Li et al., 2018). Deletion of endothelial GABA release not only disrupted the tangential migration of interneurons, but also increased the number of proliferating progenitor cells in the SVZ of the ventral telencephalon. In addition, RNA sequencing revealed dysregulation of crucial neurogenesis-related genes when endothelial-specific GABA secretion was deleted embryonically (Li et al., 2018).

Javaherian and Kriegstein observed that IPCs, which express the marker Tbr2, were preferentially distributed along the developing blood vessels in the SVZ. Via VEGF-overexpression after *in utero* electroporation, they induced the overgrowth of blood vessels and triggered the aberrant migration of Tbr2^+^ cells towards the ectopic blood vessels. Moreover, mitotic progenitors were preferentially located to branch points, where tip cells are present during branching morphogenesis (Javaherian and Kriegstein, 2009). Tip cells are specialized endothelial cells that extend filopodia to sense migratory guidance cues in their environment and mediate new contacts (Gerhardt et al., 2003). These findings suggested that IPC interact with blood vessels by contacting tip cells. Ten years later, Di Marco *et al.* elegantly confirmed this hypothesis by describing direct contacts between vascular tip cell filopodia and apical neural progenitors of the lateral ganglionic eminence in both mouse and human embryos (Figure 2C). In the same study, and thanks to series of cell birth-dating experiments in mouse models with enriched and depleted vascular filopodia, endothelial cell filopodia were shown to extend the mitotic phase of RGCs and this triggered an earlier neural differentiation while limiting the amplification of the pool of progenitor cells (Di Marco et al., 2020). In addition, RGCs establish direct contacts with the periventricular vasculature via their apical end-feet in the ventral telencephalon. Tan et al. reported that the anchorage of RGC end-feet to periventricular blood vessels is mediated by Integrin β1 (Tan et al., 2016). Deletion of Integrin β1 specifically in RGCs halved the anchoring of the end-feet and reduced the number of mitotic RGC in the VZ of the medial ganglionic eminence. Interestingly, Integrin β1-mediated RGC anchoring was critical in defining the proportion of parvalbumin and somatostatin interneurons, the two major types of neocortical interneurons (Tan et al., 2016). Thus, the vasculature of the neurogenic niche is able to regulate the proliferation state of RGCs via direct cell-cell contacts. Moreover, Integrin β1 is also required for the attachment of basal RGC processes to the pial surface (Graus-Porta et al., 2001) by binding to laminins on the meningeal surface (Radakovits et al., 2009). While anchoring of RGC end-feet to pial vessels is not required for RGC proliferation, it is crucial for radial migration of excitatory neurons and possibly their differentiation (Haubst et al., 2006). Consistent with this, deletion of *Dab1* in endothelial cells impaired the deposition of Laminin-α4 on the vasculature, which disrupted the binding of RGC processes via Integrin β1 and, consequently, altered the proper positioning of pyramidal neurons in the neocortical layers (Segarra et al., 2018).

At late embryonic stage the VZ decreases in size while the SVZ expands, and this increase continues perinatally (Brazel et al., 2003). The SVZ located at the anterior part of the lateral ventricle gives rise to neuroblasts that migrate along the rostral migratory stream to the olfactory bulbs. Remarkably, neuroblasts generated postnatally in the SVZ prematurely leave the rostral migratory stream and migrate towards the cortex using cortical blood vessels as scaffolds. In this way, a fraction of GABAergic interneurons is added to the lower cortical layers (Le Magueresse et al., 2012). Moreover, at early postnatal stages vessels progressively align longitudinally along the developing rostral migratory stream and, interestingly, neuroblast proliferation was significantly associated with the vicinity of vessels (Nie et al., 2010).

The meninges, which are initially vascularized by the PNVP and become highly irrigated by the leptomeningeal vessels during development, support the tangential migration of the Cajal-Retzius cells via CXCL12/CXCR4 interactions during embryonic development (Borrell and Marín, 2006). In addition to providing extracellular matrix components, metabolites, and growth factors that regulate neurogenesis (Siegenthaler et al., 2009; Choe et al., 2012), meninges have been shown to harbor cells that express neural precursor markers during development, suggesting that meninges may themselves represent a neurogenic niche (Bifari et al., 2015; Nakagomi and Matsuyama, 2017). Neuronal progenitors in the meninges are generated during embryonic development. They have characteristics resembling RGCs and migrate perinatally into the brain parenchyma where they differentiate into cortical neurons (Bifari et al., 2017). These meningeal neuronal progenitors migrate from the leptomeninges through the meningeal substructures below the hippocampus towards the lateral ventricle. The meningeal-derived neuroblasts maintain a close association with the vasculature during this journey, although a direct signaling from the vasculature remains to be elucidated.

## Concluding Remarks

Neurogenesis is the driving force behind CNS development. This process does not only respond to intrinsic signals from neuronal progenitors but it is also governed by the influence of the cellular milieu in the germinal zones, of which the endothelial cells are an important component. Indeed, several animal models with vascular deficits support the notion that perturbations in the vasculature have an impact on the neurogenic process. Hypoxia produced by insufficient vascularization modulates the expansion versus the differentiation of the pool of progenitors. Interestingly, vessels also exert an active role in neurogenesis, either by directly contacting neuronal progenitors or by releasing factors that modulate neurogenesis. A spatiotemporal analysis of putative molecular players in the course of neurogenesis would be relevant since unique pathways can be involved in different neurogenic niches throughout brain development. Furthermore, vascular heterogeneity could play a role in directing neurogenesis, considering that endothelial cells from PVP and PVNP express different genes (Won et al., 2013), and even transcriptional differences were found among dorsal and ventral vessels from the PVP (Vasudevan et al., 2008).

Furthermore, vessels act as conduits of blood-borne substances. These substances can reach the neurogenic niches if they are permeable to the blood-brain barrier, which is formed at embryonic stages (Daneman et al., 2010). Moreover, the choroid plexus is a vascularized structure that develops in the ventricles concomitantly to developmental neurogenesis. The choroid plexus releases molecules into the embryonic cerebrospinal fluid (CSF), which is known to contain a myriad of factors involved in neurogenesis (see review (Fame and Lehtinen, 2020)). These molecules have to cross the blood-CSF barrier to reach the ventricles. Therefore, neurogenesis can also be regulated by selective transport of molecules through the barriers within the CNS, however this field of research still remains poorly explored. In addition, it has to be considered that blood circulating maternal factors also influence the embryonic neurodevelopment in mammals.

All in all, these findings open the possibility that some neurodevelopmental defects may originate in the vascular system, either indirectly through deficits in oxygen and molecule delivery, as observed in preterm infants, or through direct endothelial-mediated signaling. In this regard, it has been observed that prematurely born rabbits exhibit an excessive pool of interneuron progenitors in the ganglionic eminence and this can be reversed by treatment with the blood-borne hormone estrogen (Tibrewal et al., 2018). Moreover, Zika virus infection in mice has been shown to cause defects in angiogenesis that are concomitant with abnormal brain development (Garcez et al., 2018). These examples suggest that the vasculature could be envisaged as a target as well as a vehicle to pharmacologically treat some neurodevelopmental disorders.

Although some advances have demonstrated that the vasculature plays a relevant role in various steps along the neurogenic process by influencing neuroblast proliferation, differentiation and migration, the molecular portfolio that orchestrates this communication between the nervous and the vascular systems remains rather elusive. Novel technologies based on omics studies as well as refined gene editing approaches will certainly contribute to unveiling these molecular players, and thus potential therapeutic targets, in the near future.
